# Tetra­aqua­bis(isonicotinamide-κ*N* ^1^)cobalt(II) bis­(4-formyl­benzoate) dihydrate

**DOI:** 10.1107/S1600536809033200

**Published:** 2009-08-26

**Authors:** Tuncer Hökelek, Filiz Yılmaz, Barış Tercan, Mustafa Sertçelik, Hacali Necefoğlu

**Affiliations:** a Department of Physics, Hacettepe University, 06800 Beytepe, Ankara, Turkey; b Department of Chemistry, Faculty of Science, Anadolu University, 26470 Yenibağlar, Eskişehir, Turkey; c Department of Physics, Karabük University, 78050 Karabük, Turkey; d Department of Chemistry, Kafkas University, 63100 Kars, Turkey

## Abstract

The asymmetric unit of the crystal structure of the title complex, [Co(C_6_H_6_N_2_O)_2_(H_2_O)_4_](C_8_H_5_O_3_)_2_·2H_2_O, contains one-half of the complex cation with the Co^II^ ion located on an inversion center, a 4-formyl­benzoate (FB) counter-anion and an uncoordinated water mol­ecule. The four O atoms in the equatorial plane around the Co^II^ ion form a slightly distorted square-planar arrangement with an average Co—O bond length of 2.086 Å; the slightly distorted octa­hedral coordination is completed by the two N atoms of the isonicotinamide (INA) ligands at a slightly longer distance [2.1603 (14) Å] in the axial positions. The dihedral angle between the carboxyl­ate group and the attached benzene ring is 5.93 (13)°, while the pyridine and benzene rings are oriented at a dihedral angle of 3.09 (6)°. In the crystal structure, O—H⋯O, N—H⋯O and C—H⋯O hydrogen bonds link the mol­ecules into a three-dimensional network. π–π Contacts between the benzene and pyridine rings [centroid–centroid distance = 3.758 (1) Å] may further stabilize the crystal structure.

## Related literature

For general background to transition metal complexes of nicotinamide and/or the nicotinic acid derivative *N*,*N*-diethyl­nicotinamide, see: Bigoli *et al.* (1972 ▶); Krishnamachari (1974 ▶). For related structures, see: Hökelek *et al.* (2009 ▶); Sertçelik *et al.* (2009 ▶).
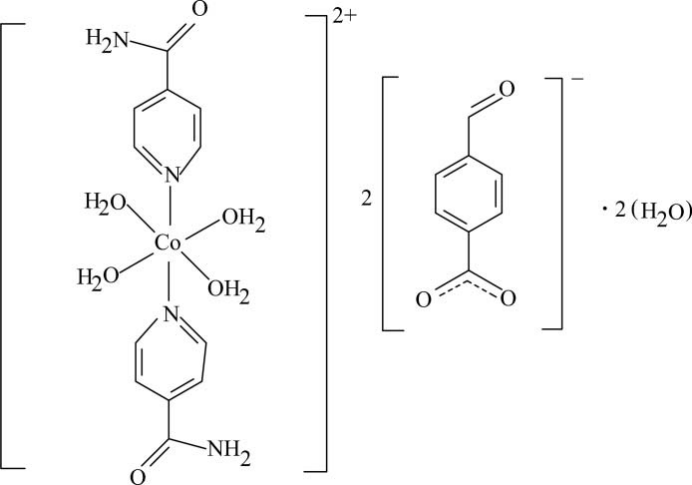


## Experimental

### 

#### Crystal data

[Co(C_6_H_6_N_2_O)_2_(H_2_O)_4_](C_8_H_5_O_3_)_2_·2H_2_O
*M* *_r_* = 709.53
Triclinic, 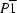

*a* = 6.4490 (2) Å
*b* = 6.8836 (3) Å
*c* = 18.1792 (5) Å
α = 81.967 (3)°
β = 84.681 (3)°
γ = 72.564 (2)°
*V* = 761.28 (5) Å^3^
*Z* = 1
Mo *K*α radiation
μ = 0.64 mm^−1^
*T* = 100 K
0.17 × 0.08 × 0.04 mm

#### Data collection

Bruker Kappa APEXII CCD area-detector diffractometer
Absorption correction: multi-scan (*SADABS*; Bruker, 2005 ▶) *T*_min_ = 0.943, *T*_max_ = 0.978
13740 measured reflections
3780 independent reflections
2884 reflections with *I* > 2σ(*I*)
*R*_int_ = 0.054

#### Refinement

*R*[*F* ^2^ > 2σ(*F* ^2^)] = 0.034
*wR*(*F* ^2^) = 0.074
*S* = 0.98
3780 reflections
236 parameters
9 restraints
H atoms treated by a mixture of independent and constrained refinement
Δρ_max_ = 0.36 e Å^−3^
Δρ_min_ = −0.41 e Å^−3^

### 

Data collection: *APEX2* (Bruker, 2007 ▶); cell refinement: *SAINT* (Bruker, 2007 ▶); data reduction: *SAINT*; program(s) used to solve structure: *SHELXS97* (Sheldrick, 2008 ▶); program(s) used to refine structure: *SHELXL97* (Sheldrick, 2008 ▶); molecular graphics: *Mercury* (Macrae *et al.*, 2006 ▶); software used to prepare material for publication: *WinGX* (Farrugia, 1999 ▶) and *PLATON* (Spek, 2009 ▶).

## Supplementary Material

Crystal structure: contains datablocks I, global. DOI: 10.1107/S1600536809033200/xu2594sup1.cif

Structure factors: contains datablocks I. DOI: 10.1107/S1600536809033200/xu2594Isup2.hkl

Additional supplementary materials:  crystallographic information; 3D view; checkCIF report

## Figures and Tables

**Table 1:** Selected bond lengths (Å)

Co1—O5	2.0570 (13)
Co1—O6	2.1151 (12)
Co1—N1	2.1603 (14)

**Table 2:** Hydrogen-bond geometry (Å, °)

*D*—H⋯*A*	*D*—H	H⋯*A*	*D*⋯*A*	*D*—H⋯*A*
N2—H2*A*⋯O4^i^	0.86	2.13	2.9680 (19)	166
N2—H2*B*⋯O7^ii^	0.86	2.05	2.8659 (19)	158
O5—H51⋯O1^iii^	0.916 (18)	1.723 (18)	2.6355 (18)	174.5 (18)
O5—H52⋯O2	0.868 (15)	1.863 (15)	2.7236 (18)	171.1 (18)
O6—H61⋯O1^iv^	0.899 (15)	1.872 (15)	2.7707 (19)	177.3 (11)
O6—H62⋯O2^v^	0.87 (2)	1.92 (2)	2.7760 (19)	168 (2)
O7—H71⋯O3^vi^	0.891 (19)	1.850 (18)	2.7371 (19)	173.7 (18)
O7—H72⋯O4^i^	0.936 (15)	1.982 (15)	2.9168 (19)	176.8 (17)
C9—H9⋯O7^ii^	0.93	2.56	3.458 (2)	161
C12—H12⋯O5^vii^	0.93	2.45	3.210 (2)	139

Symmetry codes: (i) 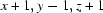; (ii) 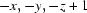; (iii) 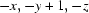; (iv) 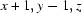; (v) 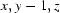; (vi) 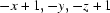; (vii) 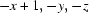.

**Fig. 1.**
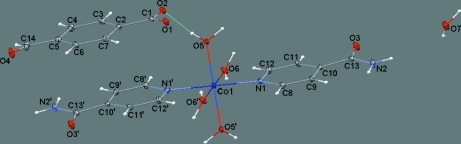
The structure of the title molecule with the atom-numbering scheme. Displacement ellipsoids are drawn at the 50% probability level. Primed atoms are generated by the symmetry operator:(') -x, -y, -z. Dashed line indicates the hydrogen-bonding.

## supplementary crystallographic information

### Comment

As a part of our ongoing investigation on transition metal complexes of nicotinamide (NA), one form of niacin (Krishnamachari, 1974), and/or the nicotinic acid derivative *N*,*N*-diethylnicotinamide (DENA), an important respiratory stimulant (Bigoli *et al.*, 1972), the title compound was synthesized and its crystal structure is reported herein.

The title compound is a monomeric complex, with Co^II^ ion on a centre of symmetry, consisting of two INA ligands, four coordinated water molecules, two FB anions and two uncoordinated water molecules. The structures of some DENA and/or INA complexes of Co^II^ ion, [Co(C_8_H_5_O_3_)_2_(C_10_H_14_N_2_O)_2_(H_2_O)_2_] (Sertçelik *et al.*, 2009) and [Co(C_6_H_6_N_2_O)(C_9_H_10_NO_2_)_2_(H_2_O)_2_] (Hökelek *et al.*, 2009) have also been determined.

In the title compound, INA ligands are monodentate. The four O atoms (O5, O6, and the symmetry-related atoms, O5', O6') in the equatorial plane around the Co atom form a slightly distorted square-planar arrangement, while the slightly distorted octahedral coordination is completed by the two pyridine N atoms (N1, N1') of the INA ligands at 2.1603 (14) Å from the Co atom in the axial positions (Table 1, Fig. 1). The average Co—O bond length is 2.0861 (13) Å. The O—H···O hydrogen bond (Table 2) links the coordinated water molecule to the FB anion. The dihedral angle between the planar carboxylate group (O1/O2/C1) and the benzene ring A (C2—C7) is 5.93 (13)°, while that between rings A and B (N1/C8—C12) is 3.09 (6)°.

In the crystal structure, O—H···O, N—H···O and C—H···O hydrogen bonds (Table 2) link the molecules into a three-dimensional network, in which they may be effective in the stabilization of the structure. The π–π contact between the benzene and pyridine rings, *Cg*1—*Cg*2, [where *Cg*1 and *Cg*2 are centroids of the rings A (C2—C7) and B (N1/C8—C12), respectively] may further stabilize the structure, with centroid-centroid distance of 3.758 (1) Å.

### Experimental

The title compound was prepared by the reaction of CoSO_4_.7H_2_O (1.41 g, 5 mmol) in H_2_O (25 ml) and INA (1.22 g, 10 mmol) in H_2_O (40 ml) with sodium 4-formylbenzoate (1.72 g, 10 mmol) in H_2_O (50 ml). The mixture was filtered and set aside to crystallize at ambient temperature for several days, giving orange single crystals.

### Refinement

Atoms H51, H52, H61, H62, H71 and H72 (for H_2_O) and H14 (for CH) were located in difference Fourier map and refined isotropically, with restrains of O5—H51 = 0.916 (13), O5—H52 = 0.865 (14), O6—H61 = 0.899 (13), O6—H62 = 0.871 (14), O7—H71 = 0.892 (14), O7—H72 = 0.935 (13) Å and H51—O5—H52 = 106.6 (16), H61—O6—H62 = 106.4 (16), H71—O7—H72 = 106.5 (15) °. The remaining H atoms were positioned geometrically with N—H = 0.86 Å (for NH_2_) and C—H = 0.93 Å, for aromatic H atoms and constrained to ride on their parent atoms, with *U*_iso_(H) = 1.2*U*_eq_(C,*N*).

### Crystal data

**Table:**

[Co(C_6_H_6_N_2_O)_2_(H_2_O)_4_](C_8_H_5_O_3_)_2_·2H_2_O	*Z* = 1
*M**_r_* = 709.53	*F*(000) = 369
Triclinic, *P*1	*D*_x_ = 1.548 Mg m^−^^3^
Hall symbol: -P 1	Mo *K*α radiation, λ = 0.71073 Å
*a* = 6.4490 (2) Å	Cell parameters from 4050 reflections
*b* = 6.8836 (3) Å	θ = 1.1–28.4°
*c* = 18.1792 (5) Å	µ = 0.64 mm^−^^1^
α = 81.967 (3)°	*T* = 100 K
β = 84.681 (3)°	Rod-shaped, orange
γ = 72.564 (2)°	0.17 × 0.08 × 0.04 mm
*V* = 761.28 (5) Å^3^	

### Data collection

**Table:**

Bruker Kappa APEXII CCD area-detector diffractometer	3780 independent reflections
Radiation source: fine-focus sealed tube	2884 reflections with *I* > 2σ(*I*)
graphite	*R*_int_ = 0.054
φ and ω scans	θ_max_ = 28.4°, θ_min_ = 1.1°
Absorption correction: multi-scan (*SADABS*; Bruker, 2005)	*h* = −8→8
*T*_min_ = 0.943, *T*_max_ = 0.978	*k* = −9→8
13740 measured reflections	*l* = −24→24

### Refinement

**Table:**

Refinement on *F*^2^	Primary atom site location: structure-invariant direct methods
Least-squares matrix: full	Secondary atom site location: difference Fourier map
*R*[*F*^2^ > 2σ(*F*^2^)] = 0.034	Hydrogen site location: inferred from neighbouring sites
*wR*(*F*^2^) = 0.074	H atoms treated by a mixture of independent and constrained refinement
*S* = 0.98	*w* = 1/[σ^2^(*F*_o_^2^) + (0.0308*P*)^2^] where *P* = (*F*_o_^2^ + 2*F*_c_^2^)/3
3780 reflections	(Δ/σ)_max_ < 0.001
236 parameters	Δρ_max_ = 0.36 e Å^−^^3^
9 restraints	Δρ_min_ = −0.41 e Å^−^^3^

### Special details

**Table:**

Geometry. All e.s.d.'s (except the e.s.d. in the dihedral angle between two l.s. planes) are estimated using the full covariance matrix. The cell e.s.d.'s are taken into account individually in the estimation of e.s.d.'s in distances, angles and torsion angles; correlations between e.s.d.'s in cell parameters are only used when they are defined by crystal symmetry. An approximate (isotropic) treatment of cell e.s.d.'s is used for estimating e.s.d.'s involving l.s. planes.
Refinement. Refinement of *F*^2^ against ALL reflections. The weighted *R*-factor *wR* and goodness of fit *S* are based on *F*^2^, conventional *R*-factors *R* are based on *F*, with *F* set to zero for negative *F*^2^. The threshold expression of *F*^2^ > σ(*F*^2^) is used only for calculating *R*-factors(gt) *etc*. and is not relevant to the choice of reflections for refinement. *R*-factors based on *F*^2^ are statistically about twice as large as those based on *F*, and *R*- factors based on ALL data will be even larger.

### Fractional atomic coordinates and isotropic or equivalent isotropic displacement parameters (Å^2^)

**Table:**

	*x*	*y*	*z*	*U*_iso_*/*U*_eq_	
Co1	0.0000	0.0000	0.0000	0.01071 (11)	
O1	−0.3206 (2)	0.68183 (19)	−0.08763 (7)	0.0168 (3)	
O2	0.0316 (2)	0.55083 (19)	−0.12091 (7)	0.0154 (3)	
O3	0.6316 (2)	−0.2347 (2)	0.31750 (7)	0.0191 (3)	
O4	−0.5540 (2)	0.7430 (2)	−0.46372 (7)	0.0249 (3)	
O5	0.1816 (2)	0.2025 (2)	−0.02613 (7)	0.0129 (3)	
H51	0.226 (3)	0.251 (3)	0.0122 (9)	0.015*	
H52	0.122 (3)	0.309 (2)	−0.0568 (9)	0.015*	
O6	0.2398 (2)	−0.2399 (2)	−0.04719 (7)	0.0154 (3)	
H61	0.382 (2)	−0.268 (3)	−0.0613 (10)	0.015*	
H62	0.189 (3)	−0.321 (3)	−0.0685 (10)	0.015*	
O7	0.1474 (2)	0.1218 (2)	0.58230 (7)	0.0212 (3)	
H71	0.222 (3)	0.165 (3)	0.6122 (9)	0.015*	
H72	0.239 (3)	−0.002 (2)	0.5679 (10)	0.015*	
N1	0.1381 (2)	−0.0933 (2)	0.10768 (8)	0.0118 (3)	
N2	0.3064 (2)	−0.1744 (2)	0.38109 (8)	0.0159 (4)	
H2A	0.3647	−0.1901	0.4230	0.019*	
H2B	0.1675	−0.1461	0.3796	0.019*	
C1	−0.1665 (3)	0.6195 (3)	−0.13455 (10)	0.0133 (4)	
C2	−0.2240 (3)	0.6309 (3)	−0.21448 (10)	0.0119 (4)	
C3	−0.0588 (3)	0.5837 (3)	−0.26939 (10)	0.0149 (4)	
H3	0.0859	0.5438	−0.2570	0.018*	
C4	−0.1087 (3)	0.5957 (3)	−0.34277 (10)	0.0164 (4)	
H4	0.0023	0.5630	−0.3795	0.020*	
C5	−0.3246 (3)	0.6567 (3)	−0.36142 (10)	0.0150 (4)	
C6	−0.4913 (3)	0.7031 (3)	−0.30633 (10)	0.0146 (4)	
H6	−0.6359	0.7427	−0.3187	0.018*	
C7	−0.4407 (3)	0.6900 (3)	−0.23320 (10)	0.0140 (4)	
H7	−0.5517	0.7206	−0.1964	0.017*	
C8	0.0137 (3)	−0.0686 (3)	0.17118 (10)	0.0130 (4)	
H8	−0.1367	−0.0279	0.1683	0.016*	
C9	0.0981 (3)	−0.1008 (3)	0.24058 (10)	0.0128 (4)	
H9	0.0057	−0.0807	0.2830	0.015*	
C10	0.3216 (3)	−0.1633 (3)	0.24610 (10)	0.0114 (4)	
C11	0.4511 (3)	−0.1974 (3)	0.18124 (10)	0.0137 (4)	
H11	0.6018	−0.2448	0.1830	0.016*	
C12	0.3547 (3)	−0.1602 (3)	0.11403 (10)	0.0146 (4)	
H12	0.4440	−0.1827	0.0710	0.018*	
C13	0.4314 (3)	−0.1941 (3)	0.31843 (10)	0.0132 (4)	
C14	−0.3744 (3)	0.6684 (3)	−0.43957 (11)	0.0203 (5)	
H14	−0.251 (3)	0.611 (3)	−0.4732 (11)	0.025 (6)*	

### Atomic displacement parameters (Å^2^)

**Table:**

	*U*^11^	*U*^22^	*U*^33^	*U*^12^	*U*^13^	*U*^23^
Co1	0.0088 (2)	0.0147 (2)	0.00937 (19)	−0.00406 (16)	−0.00113 (14)	−0.00213 (14)
O1	0.0131 (7)	0.0241 (8)	0.0145 (7)	−0.0058 (6)	−0.0016 (5)	−0.0053 (6)
O2	0.0110 (7)	0.0181 (7)	0.0175 (7)	−0.0034 (6)	−0.0053 (5)	−0.0027 (6)
O3	0.0116 (7)	0.0274 (8)	0.0181 (7)	−0.0028 (6)	−0.0042 (6)	−0.0068 (6)
O4	0.0248 (8)	0.0311 (9)	0.0159 (7)	−0.0013 (7)	−0.0082 (6)	−0.0036 (6)
O5	0.0124 (7)	0.0173 (7)	0.0101 (7)	−0.0054 (6)	−0.0040 (5)	−0.0007 (5)
O6	0.0100 (7)	0.0195 (8)	0.0186 (7)	−0.0049 (6)	0.0001 (6)	−0.0078 (6)
O7	0.0157 (8)	0.0304 (9)	0.0191 (7)	−0.0062 (7)	−0.0028 (6)	−0.0079 (6)
N1	0.0107 (8)	0.0134 (8)	0.0123 (8)	−0.0047 (7)	−0.0004 (6)	−0.0021 (6)
N2	0.0137 (8)	0.0243 (9)	0.0106 (8)	−0.0048 (7)	−0.0051 (6)	−0.0033 (7)
C1	0.0166 (10)	0.0101 (10)	0.0147 (9)	−0.0058 (8)	−0.0034 (8)	−0.0011 (7)
C2	0.0132 (10)	0.0095 (9)	0.0138 (9)	−0.0041 (8)	−0.0028 (7)	−0.0006 (7)
C3	0.0114 (10)	0.0148 (10)	0.0185 (10)	−0.0031 (8)	−0.0032 (8)	−0.0024 (8)
C4	0.0154 (10)	0.0163 (10)	0.0155 (10)	−0.0024 (8)	0.0017 (8)	−0.0020 (8)
C5	0.0193 (10)	0.0133 (10)	0.0126 (9)	−0.0045 (8)	−0.0025 (8)	−0.0012 (8)
C6	0.0114 (10)	0.0160 (10)	0.0166 (10)	−0.0033 (8)	−0.0042 (8)	−0.0018 (8)
C7	0.0138 (10)	0.0132 (10)	0.0146 (9)	−0.0029 (8)	−0.0001 (8)	−0.0028 (8)
C8	0.0091 (9)	0.0152 (10)	0.0140 (9)	−0.0023 (8)	−0.0018 (7)	−0.0015 (8)
C9	0.0122 (10)	0.0160 (10)	0.0104 (9)	−0.0048 (8)	0.0010 (7)	−0.0022 (7)
C10	0.0141 (10)	0.0085 (9)	0.0127 (9)	−0.0038 (8)	−0.0039 (7)	−0.0014 (7)
C11	0.0089 (9)	0.0168 (10)	0.0161 (10)	−0.0040 (8)	−0.0032 (7)	−0.0021 (8)
C12	0.0108 (10)	0.0190 (10)	0.0136 (9)	−0.0036 (8)	0.0005 (7)	−0.0028 (8)
C13	0.0147 (10)	0.0112 (10)	0.0143 (9)	−0.0031 (8)	−0.0035 (8)	−0.0027 (7)
C14	0.0236 (12)	0.0214 (11)	0.0143 (10)	−0.0039 (10)	0.0002 (9)	−0.0033 (9)

### Geometric parameters (Å, °)

**Table:**

Co1—O5	2.0570 (13)	C2—C3	1.387 (2)
Co1—O5^i^	2.0570 (13)	C3—C4	1.387 (2)
Co1—O6	2.1151 (12)	C3—H3	0.9300
Co1—O6^i^	2.1151 (12)	C4—H4	0.9300
Co1—N1	2.1603 (14)	C5—C4	1.388 (3)
Co1—N1^i^	2.1603 (14)	C5—C14	1.471 (3)
O1—C1	1.261 (2)	C6—C5	1.394 (2)
O2—C1	1.257 (2)	C6—C7	1.382 (2)
O3—C13	1.236 (2)	C6—H6	0.9300
O4—C14	1.214 (2)	C7—C2	1.394 (2)
O5—H51	0.916 (13)	C7—H7	0.9300
O5—H52	0.865 (14)	C8—H8	0.9300
O6—H61	0.899 (13)	C9—C8	1.383 (2)
O6—H62	0.871 (14)	C9—C10	1.384 (2)
O7—H71	0.892 (14)	C9—H9	0.9300
O7—H72	0.935 (13)	C10—C11	1.386 (2)
N1—C8	1.344 (2)	C10—C13	1.510 (2)
N1—C12	1.344 (2)	C11—C12	1.380 (2)
N2—C13	1.331 (2)	C11—H11	0.9300
N2—H2A	0.8600	C12—H12	0.9300
N2—H2B	0.8600	C14—H14	0.976 (19)
C2—C1	1.518 (2)		
			
O5—Co1—O5^i^	180.00 (4)	C4—C3—C2	120.16 (17)
O5—Co1—O6	93.11 (5)	C4—C3—H3	119.9
O5^i^—Co1—O6	86.89 (5)	C3—C4—C5	119.95 (17)
O5—Co1—O6^i^	86.89 (5)	C3—C4—H4	120.0
O5^i^—Co1—O6^i^	93.11 (5)	C5—C4—H4	120.0
O5—Co1—N1	90.50 (5)	C4—C5—C6	120.09 (16)
O5^i^—Co1—N1	89.50 (5)	C4—C5—C14	119.15 (17)
O5—Co1—N1^i^	89.50 (5)	C6—C5—C14	120.75 (17)
O5^i^—Co1—N1^i^	90.50 (5)	C5—C6—H6	120.1
O6—Co1—O6^i^	180.00 (9)	C7—C6—C5	119.78 (17)
O6—Co1—N1	91.81 (5)	C7—C6—H6	120.1
O6^i^—Co1—N1	88.19 (5)	C2—C7—H7	119.9
O6—Co1—N1^i^	88.19 (5)	C6—C7—C2	120.21 (17)
O6^i^—Co1—N1^i^	91.81 (5)	C6—C7—H7	119.9
N1^i^—Co1—N1	180.00 (3)	N1—C8—C9	123.34 (16)
Co1—O5—H51	118.1 (12)	N1—C8—H8	118.3
Co1—O5—H52	114.2 (12)	C9—C8—H8	118.3
H51—O5—H52	106.5 (16)	C8—C9—C10	119.24 (16)
Co1—O6—H61	136.9 (12)	C8—C9—H9	120.4
Co1—O6—H62	114.8 (12)	C10—C9—H9	120.4
H62—O6—H61	106.4 (16)	C9—C10—C11	117.81 (16)
H71—O7—H72	106.5 (15)	C9—C10—C13	123.74 (16)
C8—N1—Co1	121.76 (12)	C11—C10—C13	118.44 (16)
C12—N1—Co1	121.13 (12)	C10—C11—H11	120.3
C12—N1—C8	116.80 (15)	C12—C11—C10	119.46 (17)
C13—N2—H2A	120.0	C12—C11—H11	120.3
C13—N2—H2B	120.0	N1—C12—C11	123.26 (16)
H2A—N2—H2B	120.0	N1—C12—H12	118.4
O2—C1—O1	125.45 (16)	C11—C12—H12	118.4
O2—C1—C2	117.12 (16)	O3—C13—N2	122.54 (16)
O1—C1—C2	117.42 (16)	O3—C13—C10	119.36 (16)
C3—C2—C1	119.50 (16)	N2—C13—C10	118.09 (16)
C3—C2—C7	119.82 (16)	O4—C14—C5	124.77 (19)
C7—C2—C1	120.68 (16)	O4—C14—H14	119.5 (11)
C2—C3—H3	119.9	C5—C14—H14	115.7 (11)
			
O5—Co1—N1—C8	119.00 (13)	C6—C5—C4—C3	−0.9 (3)
O5^i^—Co1—N1—C8	−61.00 (13)	C14—C5—C4—C3	−179.95 (17)
O5—Co1—N1—C12	−54.41 (14)	C4—C5—C14—O4	−170.50 (19)
O5^i^—Co1—N1—C12	125.59 (14)	C6—C5—C14—O4	10.4 (3)
O6—Co1—N1—C8	−147.87 (13)	C7—C6—C5—C4	0.6 (3)
O6^i^—Co1—N1—C8	32.13 (13)	C7—C6—C5—C14	179.65 (17)
O6—Co1—N1—C12	38.72 (14)	C5—C6—C7—C2	0.1 (3)
O6^i^—Co1—N1—C12	−141.28 (14)	C6—C7—C2—C1	178.94 (16)
Co1—N1—C8—C9	−171.09 (13)	C6—C7—C2—C3	−0.5 (3)
C12—N1—C8—C9	2.6 (3)	C10—C9—C8—N1	−0.6 (3)
Co1—N1—C12—C11	171.72 (14)	C8—C9—C10—C11	−2.1 (3)
C8—N1—C12—C11	−2.0 (3)	C8—C9—C10—C13	177.26 (16)
C3—C2—C1—O1	173.53 (16)	C9—C10—C11—C12	2.6 (3)
C3—C2—C1—O2	−5.6 (2)	C13—C10—C11—C12	−176.75 (16)
C7—C2—C1—O1	−5.9 (3)	C9—C10—C13—O3	−174.44 (17)
C7—C2—C1—O2	174.98 (16)	C9—C10—C13—N2	5.0 (3)
C1—C2—C3—C4	−179.23 (16)	C11—C10—C13—O3	4.9 (3)
C7—C2—C3—C4	0.2 (3)	C11—C10—C13—N2	−175.59 (16)
C2—C3—C4—C5	0.5 (3)	C10—C11—C12—N1	−0.6 (3)

Symmetry codes: (i) −*x*, −*y*, −*z*.

### Hydrogen-bond geometry (Å, °)

**Table:**

*D*—H···*A*	*D*—H	H···*A*	*D*···*A*	*D*—H···*A*
N2—H2A···O4^ii^	0.86	2.13	2.9680 (19)	166
N2—H2B···O7^iii^	0.86	2.05	2.8659 (19)	158
O5—H51···O1^iv^	0.92 (2)	1.72 (2)	2.6355 (18)	175 (2)
O5—H52···O2	0.87 (2)	1.86 (2)	2.7236 (18)	171 (2)
O6—H61···O1^v^	0.90 (2)	1.87 (2)	2.7707 (19)	177 (1)
O6—H62···O2^vi^	0.87 (2)	1.92 (2)	2.7760 (19)	168 (2)
O7—H71···O3^vii^	0.89 (2)	1.85 (2)	2.7371 (19)	174 (2)
O7—H72···O4^ii^	0.94 (2)	1.98 (2)	2.9168 (19)	177 (2)
C9—H9···O7^iii^	0.93	2.56	3.458 (2)	161
C12—H12···O5^viii^	0.93	2.45	3.210 (2)	139

Symmetry codes: (ii) *x*+1, *y*−1, *z*+1; (iii) −*x*, −*y*, −*z*+1; (iv) −*x*, −*y*+1, −*z*; (v) *x*+1, *y*−1, *z*; (vi) *x*, *y*−1, *z*; (vii) −*x*+1, −*y*, −*z*+1; (viii) −*x*+1, −*y*, −*z*.
